# Quercetin Influences Quorum Sensing in Food Borne Bacteria: *In-Vitro* and *In-Silico* Evidence

**DOI:** 10.1371/journal.pone.0134684

**Published:** 2015-08-06

**Authors:** Venkadesaperumal Gopu, Chetan Kumar Meena, Prathapkumar Halady Shetty

**Affiliations:** 1 Department of Food science and Technology, Pondicherry University, Pondicherry, India; 2 Department of Bio-informatics, Pondicherry University, Pondicherry, India; Shanghai Jiao Tong University, CHINA

## Abstract

Quorum sensing (QS) plays a vital role in regulating the virulence factor of many food borne pathogens, which causes severe public health risk. Therefore, interrupting the QS signaling pathway may be an attractive strategy to combat microbial infections. In the current study QS inhibitory activity of quercetin and its anti-biofilm property was assessed against food-borne pathogens using a bio-sensor strain. In addition *in-silico* techniques like molecular docking and molecular dynamics simulation studies were applied to screen the quercetin’s potentiality as QS inhibitor. Quercetin (80μg/ml) showed the significant reduction in QS-dependent phenotypes like violacein production, biofilm formation, exopolysaccharide (EPS) production, motility and alginate production in a concentration-dependent manner. Synergistic activity of conventional antibiotics with quercetin enhanced the susceptibility of all tested pathogens. Furthermore, Molecular docking analysis revealed that quercetin binds more rigidly with LasR receptor protein than the signaling compound with docking score of -9.17Kcal/mol. Molecular dynamics simulation predicted that QS inhibitory activity of quercetin occurs through the conformational changes between the receptor and quercetin complex. Above findings suggest that quercetin can act as a competitive inhibitor for signaling compound towards LasR receptor pathway and can serve as a novel QS-based antibacterial/anti-biofilm drug to manage food-borne pathogens.

## Introduction

Food spoilage and biofilm formation by food-borne pathogens are significant problems in the food industry. Even with the modern food preservation techniques, the excessive amount of food lost due to microbial spoilage [1]. Food borne pathogens like *Bacillus* spp., *Pseudomonas* spp., *Salmonella* spp., *Campylobacter jejuni* and *Yersinia enterocolitica* were identified to form biofilms [2]. Attachment of these food borne pathogens to the food product or the processing surfaces leads to severe public health risk and economic losses [3]. Several biochemical processes like lipolytic and proteolytic activities associated with food deterioration are regulated by quorum sensing [4]. Hence, microbial activity is considered to be the most common cause of food spoilage [5]. In recent years, bacterial cell-to-cell communication has received attention to manifest the role of quorum signals in the attachment and growth of pathogenic bacteria in foods [1]. Identification of quorum signals in spoiled food products has added a new element to cram the process of food spoilage.

Bacterial communications are mediated through the production of small signal molecules called auto-inducers. N-Acyl homoserine lactone (AHL) is the most extensively studied autoinducer molecule in Gram-negative bacteria [6]. AHL synthesized by LuxI autoinducer homologues, binds specifically to LuxR receptor protein to trigger specific gene expressions including, virulence factor production in *P*. *aeruginosa*(LasI/R) [7]. As quorum-sensing signaling molecules are implicated in food spoilage, disrupting the bacterial signaling mechanism can play a key role in regulating microbial gene expression related to food spoilage and human infection.

Many synthetic and natural compounds have been investigated for its quorum sensing inhibitory (QSI) activity. These compounds include macrolides [8], furanones [9], ajoene [10], ellagic acid [11] and aspirin [12]. However, limitations of these compounds to use in food industries and mammalian cells have led to a search of novel QS inhibitor for their potential use in various applications. In this study, quercetin a typical flavonoid ubiquitously used in dietary supplementation known for its anti-oxidant property is screened for QS inhibitory activity. Here quercetin below to its minimum inhibitory concentration (MIC) level was tested for its activity against QS-regulated phenotypes of food-borne pathogens, particularly biofilm formation, EPS production and flagellar-mediated motility. Further to understand the mechanism of QS inhibitory activity, the *in-silico* analysis including molecular docking and simulation studies were conducted to show the conformational changes in the LasR receptor protein of *P*. *aeruginosa*.

## Materials and Methods

### Bacterial strains and culture conditions

A mutant strain of *Chromobacterium violaceum* CV026 (CECT5999) procured from Spanish type culture collection, a derivative of wild-type unable to synthesize its AHLs was used as reporter strain. *Pseudomonas aeruginosa* strain PUFSTb04 (GenBank: KR476388), *Yersinia enterocolitica* strain PUFSTb09 (GenBank: KT266804) and *Klebsiella pneumoniae* strain PUFST23(GenBank: KF817575; MTCC 12202), foodborne isolates from the departmental culture collection was used as experimental models. Bacterial strains were selected on the basis of QS dependent phenotypes. All the cultures were grown in nutrient broth, except CECT 5999 which was routinely cultured aerobically in Luria-Bertani (LB) broth supplemented with kanamycin (20μg/ml). N-Octanoyl-DL-homoserine lactone (OHL) (Sigma-Aldrich, India) was added to induce violacein production in CECT5999 when required.

### Minimal inhibitory concentration of quercetin

The stock solution was prepared by dissolving 10mg of quercetin (Himedia, India) in 1 ml of 95% methanol. The MIC of quercetin against each organism was determined as per the guidelines of clinical and laboratory standards institute, USA. Briefly, 1% of overnight grown cultures (0.4 OD@600_nm_) were added to appropriate growth medium supplemented with quercetin to attain the final concentration ranging from (1 to 250μg/ml). Microtiter plates (MTP) were incubated overnight and recorded for MIC as the lowest concentration that showed complete inhibition of visible growth. All further experiments in the present study were performed only at sub-MIC concentrations of quercetin.

### QSI bioassay

QS Inhibitory potential of quercetin was detected using *C*. *violaceum* CV026. Overnight grown culture of reporter strain was swabbed evenly into the LB agar plates supplemented with 5 μM of OHL. Twenty microliters of quercetin at different concentration (20–80 μg/ml) was loaded on to the sterile discs, dried and placed on the agar plates and incubated at 37°C. Plates were observed for violacein inhibition by an obscure, colorless, but doable halo around the discs. Dimethyl sulfoxide (DMSO) was used as a control.

Furthermore, violacein inhibition by quercetin in the reporter strain *C*. *violaceum* CV026 was quantitatively analyzed using MTP method. Briefly, 1% of overnight grown culture (0.4 OD@600_nm_) was inoculated into LB broth supplemented with 10 μM of OHL in a 12-well microtiter plate. Wells were added with quercetin at different concentration (0–80 μg/ml) and incubated at 37°C for 24 h and extracted for violacein pigment [13].

One milliliter of culture from each well was centrifuged at 13,000 rpm for 5 min to precipitate violacein. Obtained pellet was dissolved in 1 ml of DMSO and vortexed robustly to solubilize the violacein completely. The mixture was centrifuged again to remove the bacterial cells and quantified at 585nm using microplate reader (Biotek, USA). The experiment was repeated for triplicate values, and the percentage of inhibition was calculated by the formula

$$\frac{controlOD585\;nm–testOD585\;nm}{controlOD585\;nm}\times \;100$$

### Anti-biofilm activity of quercetin

The microtiter plate assay was performed to quantify the effect of quercetin on the biofilm formation of test bacterial strains [14]. LB broth with and without quercetin (5–40 μg/ml) were inoculated with 1% of bacterial cultures (0.4 OD@600_nm_) and incubated at 37°C for 24 h. After incubation, wells were carefully rinsed with double-distilled water to remove loosely attached cells. Adhered cell on the walls were stained with 100 μl of 0.2% crystal violet solution (HiMedia, India) for 10 min. Excess stain was removed by rinsing with distilled water and washed with 100 μl of 95% ethanol. Intensity was measured at OD_650nm_ by using microplate reader (Biotek, USA), for quantification of biofilm biomass.

### Effect of quercetin on exopolysaccharide production

Effect of quercetin on the exopolysaccharide (EPS) production was examined as described by Huston et al. [15]. Briefly, LB broth with and without quercetin (5–40 μg/ml) were inoculated with 1% of test bacterial cultures (0.4 OD@600_nm_) and incubated at 37°C overnight. At the end of incubation, culture tubes were centrifuged at 5000 g for 30 minutes. Filtered supernatant (0.22μm) was added to three volumes of chilled ethanol and incubated overnight at 2°C to precipitate the dislodged EPS. Precipitated EPS was collected by centrifugation at 5000 g for 30 min at 2°C, and the pellet was dissolved in 1 ml of deionized water, stored at -40°C until further use. Total carbohydrate content in the EPS was quantified by the phenol-sulfuric acid method using glucose as a standard [15].

### Alginate production

One percent, overnight culture of *P*. *aeruginosa* (0.4 OD@600_nm_) was added to nutrient broth supplemented with or without quercetin (5–40 μg/ml). Inoculated culture broths were incubated overnight at 37°C in the shaker incubator. After incubation alginate production was estimated as described by Mousavi et al. [16]. Briefly, 600 μl of boric acid-sulphuric acid solution in the ratio of 4:1 was slowly added to 70 μl of test sample placed in an ice bath. The mixture was vortexed for 10s and placed back in the ice bath. To the above mixture 20 μl of 0.2% carbazole dissolved in ethanol was added and vortexed for 10s. The mixture was then incubated at 55°C for 30 min. Alginate production was quantified at 530nm using microplate reader (Biotek, USA).

### Motility assay

Out of 3 test strains *P*. *aeruginosa* and *Y*. *enterocolitica* were selected for this study as they were found to be motile. For the swimming assay, overnight grown bacterial cultures were point inoculated at the center of swimming agar plates (1g tryptone, 0.5g NaCl and 0.3g agar/100ml) with different concentration of quercetin (0–80 μg/ml). For swarming assay, test strains were inoculated at the center of swarming agar plates (1g peptone, 0.5g NaCl, 0.5g agar and 0.5g of filter sterilized D-glucose/100ml) with different concentration of quercetin (0–80 μg/ml). Inoculated plates were incubated at 37°C for *P*. *aeruginosa* and 26°C for *Y*. *enterocolitica* for 20–24 h. Agar medium without quercetin was served as control.

### Synergistic effects of quercetin with antibiotics

Microtiter plate containing 1ml of LB broth was added with 1% test strains (0.4 OD@600_nm_) along with quercetin at the sub-MIC concentration (5, 20 and 40 μg/ml). Inoculated broth was added with antibiotics which include erythromycin (10μg), tetracycline (30μg), kanamycin (30μg), gentamycin (10μg), ampicillin (10μg), and chloramphenicol (10μg). MTP plates were incubated at 37°C for 24 h and noted for growth reduction. Respective controls were maintained for both quercetin and antibiotics [17].

### Fractional inhibitory concentration

Synergistic effect resulted from combining quercetin with antibiotics were assessed by determining fractional inhibitory concentration (FIC) index. FIC was determined by the formula: FIC index = FIC A+ FIC B = [A]/MIC A + [B]/MIC B, where [A] is the concentration of drug A, MICA is it’s MIC and FICA is the FIC of drug A for the organism, while [B], MICB, and FICB are defined in the same fashion for drug B. The FIC index thus obtained was interpreted as follows: <0.5, synergy; 0.5 to 0.75, partial synergy; 0.76 to 1.0, additive effect; >1.0 to 4.0, indifference; and >4.0, antagonism [18]. Finally, the varying rates of synergy between the agents were determined.

### Homologous sequence analysis

Out of three bacterial test strains, *K*. *pneumoniae* was found to use an AI-2 family of autoinducers, whereas *P*. *aeruginosa* and *Y*. *enterocolitica* share common class of AHL based signaling mechanism. Hence, LasR receptor protein of *P*. *aeruginosa* and YenR receptor protein of *Y*. *enterocolitica* were compared for its homology. Homology of the genes coding for the receptor protein LasR and YenR was screened through sequence alignment.

### Docking

Molecular docking analysis was performed to identify the conformational changes in the protein structure due to the interaction of quercetin with LasR receptor protein. Docking was performed individually with quercetin and signal molecule (N-Octanoyl-DL-homoserine lactone). Docking of receptor protein with both quercetin and OHL was performed to screen whether quercetin can act as a competitive inhibitor for autoinducer molecule. Three-dimensional structure of quercetin and LasR receptor protein was retrieved from DrugBank and PDB database (PDB ID: 2UV0) respectively [19]. PDB 2UV0 structure contains four chains (E, F, G, and H) whose confirmation was similar which was analyzed by superimposing with chimera. Since, the H chain is longest and contained the preferred binding site for the natural ligand, all the water molecules and other chains were removed from the LasR receptor protein. Docking analysis was performed by using prepared protein, signaling molecule and quercetin with the help of Schrodinger suite 2012.

### Molecular dynamics simulation

Followed by the docking analysis, molecular dynamics simulation studies were performed to study the conformational changes in the receptor due to binding of signaling molecule or quercetin. Protein-signaling molecule and protein-active compound complex were simulated by Gromacs4.5.3 [20] simulation package using gromos force field [21]. All the complexes were placed into a cubic box with the size of 1.2 Å along with SPCE water model as the solvent. The system was equilibrated well before final simulation of 20 ns with the time step of 2ps.

### Statistical analysis

All the experiments were repeated for triplicate values. Data represented were mean of triplicates and the differences among the control, and test samples were analyzed by using One-way repeated measures ANOVA with *p* ≤ 0.05.

## Results

### MIC of Quercetin

Minimum inhibitory concentration was determined for quercetin against biosensor strain *C*. *violaceum* and three test bacterial strains. MIC was determined as the lowest concentration that showed complete inhibition of visible growth. Quercetin exhibited growth inhibition against reporter strain as well as the test strains. The MIC of quercetin was found to be 95μg/ml for *Y*. *enterocolitica*, 120μg/ml for *C*. *violaceum*, and 80μg/ml for *P*. *aeruginosa* and *K*. *pneumoniae*. Hence, all further experiments were carried out at sub-MIC concentrations (10–80 μg/ml for reporter strain, and 5–40 μg/ml for test strains).

### QSI bioassay

**Fig 1:**
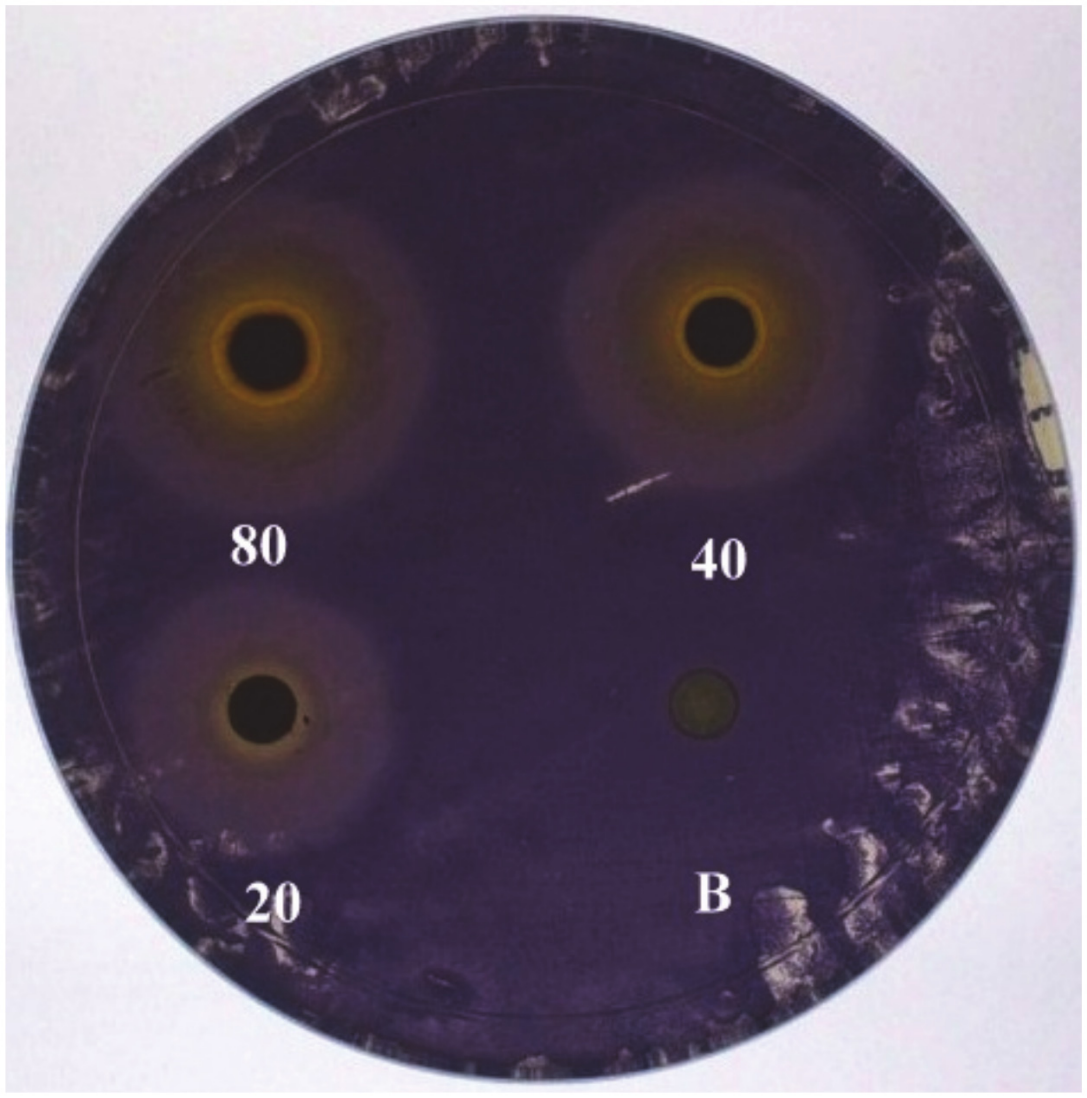
Quorum sensing bioassay of quercetin at different concentration (20, 40 and 80μg /ml) showing inhibition of OHL mediated violacein inhibition.

QS inhibitory nature of quercetin was assessed by employing *C*. *violaceum* CV026 indicated by the loss of purple pigmentation. Quercetin at all tested concentration inhibited violacein production in a concentration dependent manner. A clear inner zone represents the bacterial growth inhibition by quercetin. Whereas opaque, non-transparent halo zones around the discs represents the inhibition of violacein production, which may be due to specific interference with induced AHL production. As concentration increases, there was an increase in the size of halo zones around the discs (Fig 1).

**Fig 2:**
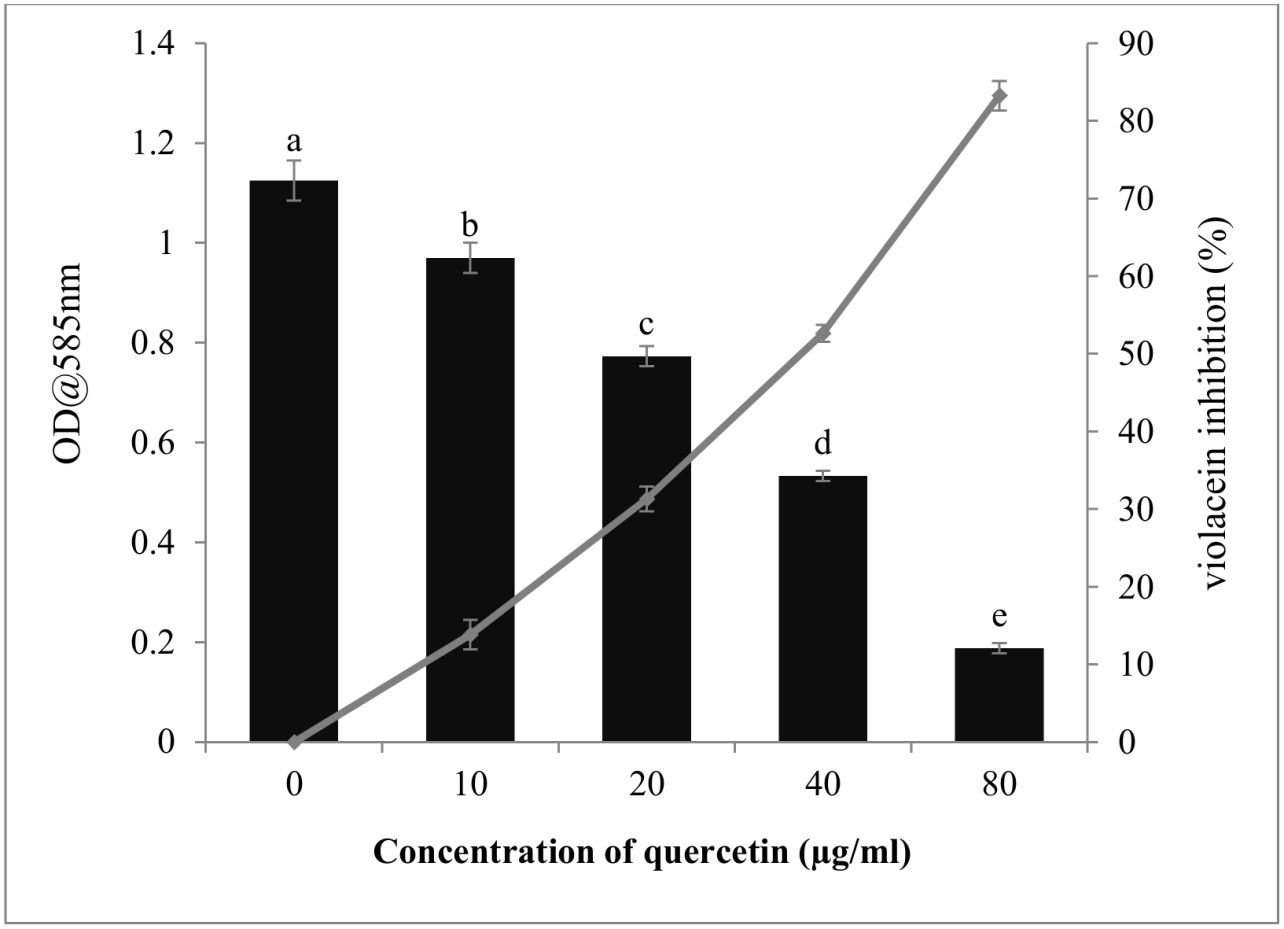
Inhibition of violacein production in *C*.*violaceum* (CECT5999)by quercetin at different concentration (0–80μg/ml). Line graph represents the percentage inhibition. Vertical bars represent the mean values of triplicates with standard deviation. Same letters in the column are not significantly different (p< 0.05).

In the quantitative determination of violacein inhibition by MTP assay, quercetin at all tested concentration exhibited a significant drop in violacein content without inhibiting bacterial growth. At the concentration of 20μg/ml quercetin inhibited violacein production up to 13.81% when compared to control (p<0.05). A gradual increase in the inhibitory activity was observed with increasing concentration of quercetin to a maximum of 83.23% at the concentration of 80μg/ml (Fig 2).

### Inhibition of biofilm formation

**Fig 3:**
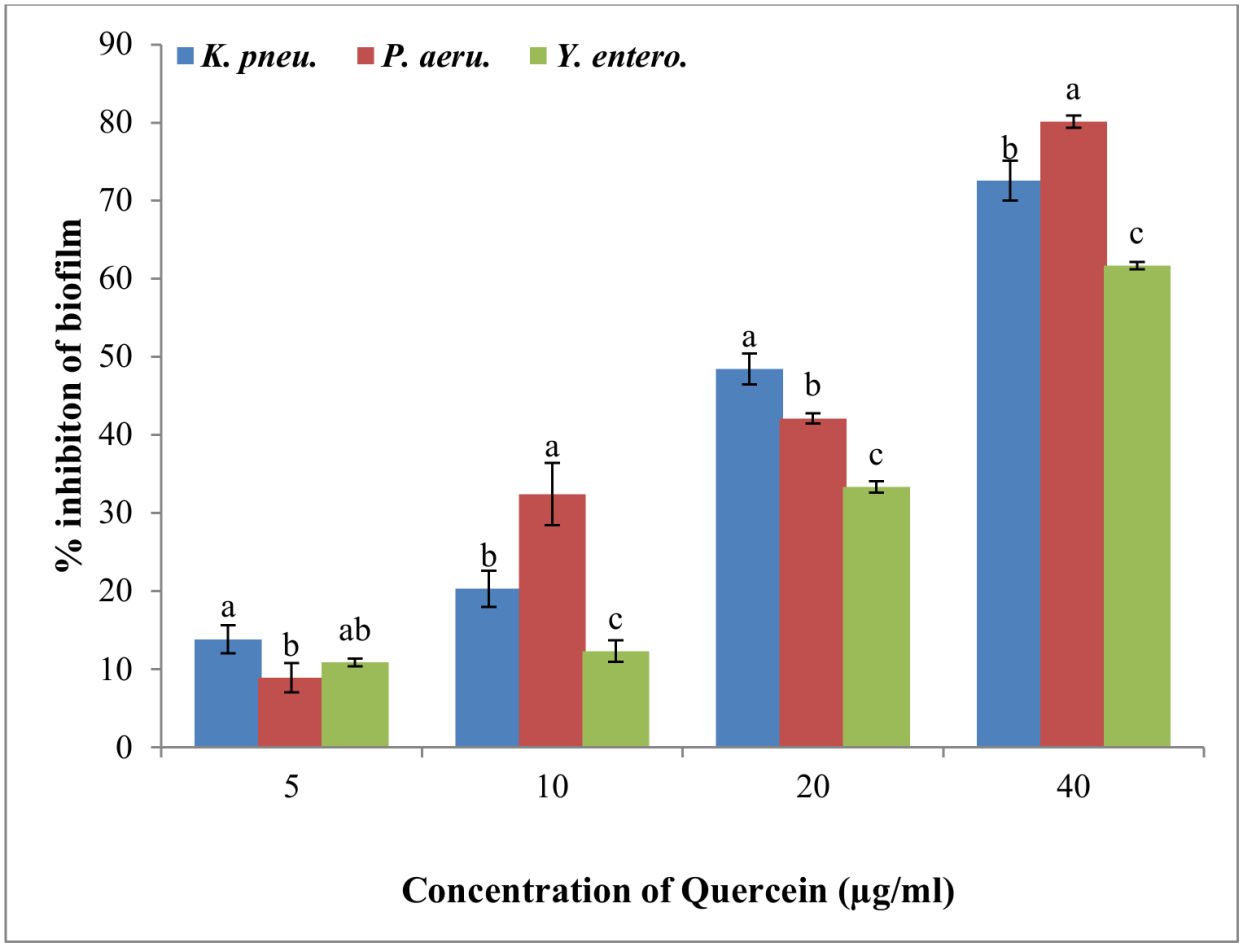
Quantitative analysis for inhibition of biofilm formation in test strains by quercetin at different concentration (0–40μg/ml). Data represented were mean of triplicate values with standard deviation. Same letters in the column are not significantly different (p< 0.05).

In the quantitative assay for screening anti-biofilm activity, quercetin exhibited a concentration-dependent reduction in the biomass of test bacterial strains. Quercetin showed 13–72%, 8–80%, and 10–61% reduction in biofilm biomass of *K*. *pneumoniae*, *P*. *aeruginosa*, and *Y*. *enterocolitica* respectively. At all tested concentration, quercetin significantly reduced biofilm biomass of all tested strains (Fig 3).

### Reduction in EPS production

**Fig 4:**
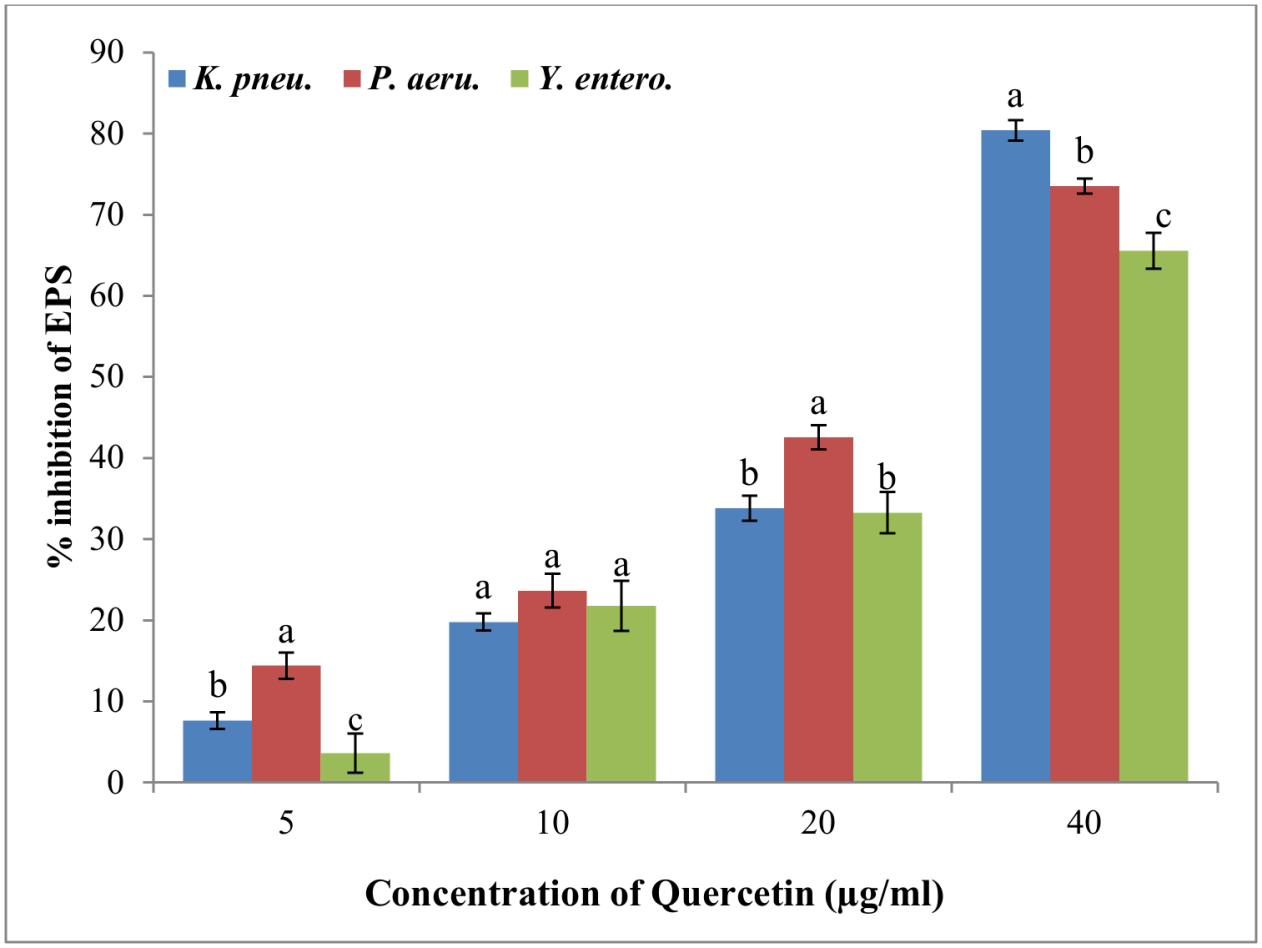
Quantitative analysis for inhibition of EPS production in test strains by quercetin at different concentration (0–40μg/ml). Data represented were mean of triplicate values with standard deviation. Same letters in the column are not significantly different (p< 0.05).

Effect of quercetin on EPS production of test bacteria was screened as it is positively correlated with biofilm formation. Quantitative analysis of EPS extracted from treated and untreated cultures revealed that EPS production decreased with increasing concentration of quercetin. Quercetin at 40μg/ml inhibited EPS production in *K*. *pneumoniae*, *P*. *aeruginosa*,and *Y*. *enterocolitica* by 80.39%, 73.52%, and 65.56% respectively (Fig 4).

### Alginate production

**Fig 5:**
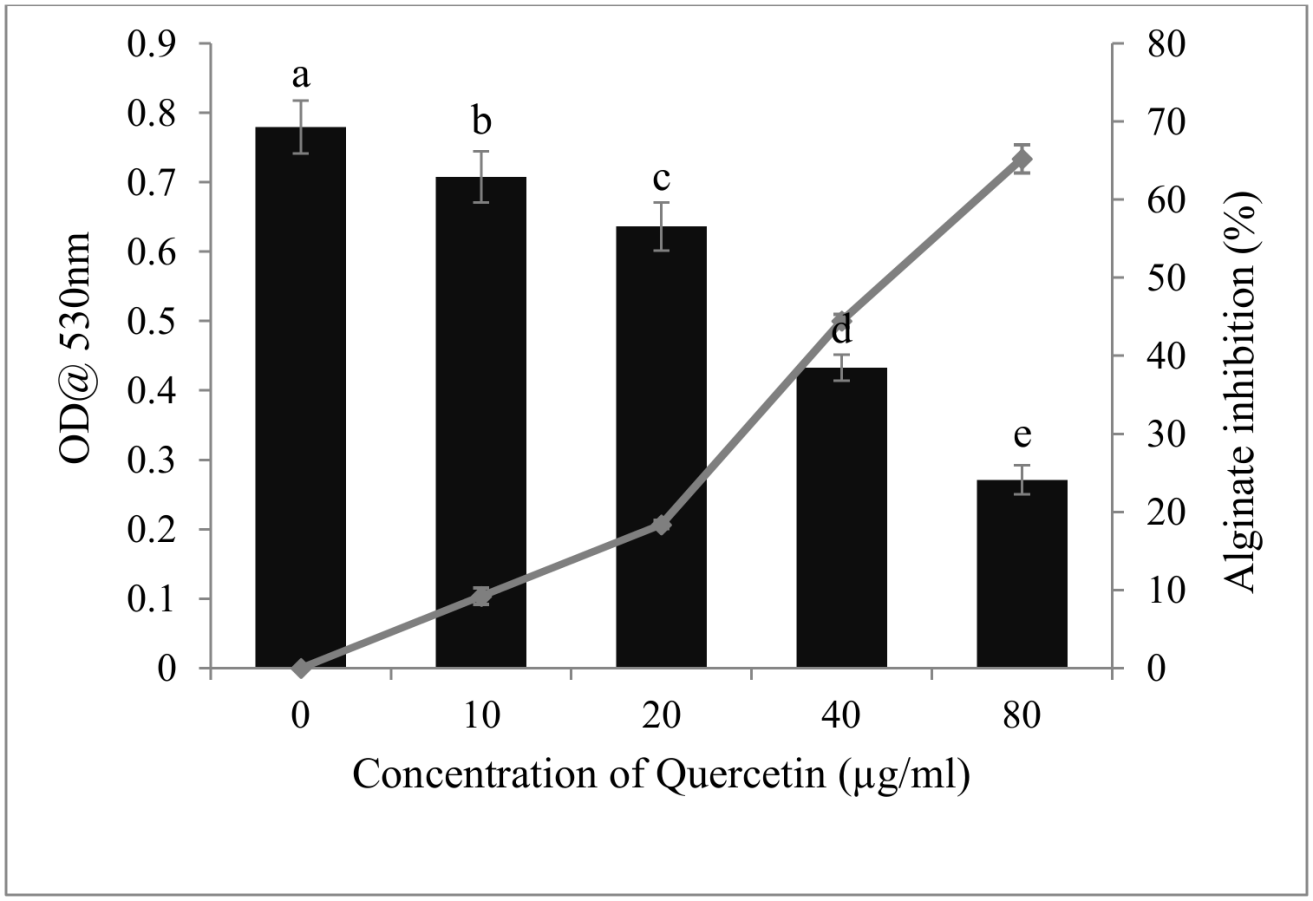
Inhibition of alginate production in *P*. *aeruginosa*by quercetin at different concentration(0–80). Mean values of triplicate independent experiments and SD are shown.

Alginate was extracted from treated and untreated cultures of test bacterial strains. Quantitative analysis revealed that the alginate production decreased with increasing concentration of quercetin. Quercetin exhibited 9–65% reduction in alginate production of *P*. *aeruginosa* at concentrations of 5–40 μg/ml (Fig 5).

### Motility assay

As flagellar mediated swimming and swarming motility plays a crucial role in QS-dependent biofilm formation, the effect of quercetin to inhibit the motility of test strains were examined. It was interesting to note that quercetin significantly inhibited the swimming and swarming behavior of *P*. *aeruginosa* and *Y*. *enterocolitica*. The higher level of inhibition was observed at the concentration of 80μg/ml (Table 1).

**Table 1:** Effect of quercetin on swimming and swarming motility of test strains.

Test Strains	Swimming motility (mm)			Swarming motility (mm)		
	0 μg/ml	40 μg/ml	80 μg/ml	0 μg/ml	40 μg/ml	80 μg/ml
*P*. *aeruginosa*	3.43 ± 0.25	2.23 ± 0.11	1.56 ± 0.20	2.83 ± 0.05	1.56 ± 0.20	0.70 ± 0.05
*Y*. *enterocolitica*	2.73 ± 0.15	1.46 ± 0.28	0.73 ± 0.05	2.66 ± 0.05	1.16 ± 0.15	0.63 ± 0.05

### Synergistic activity

The assay was performed to examine the synergistic activity of quercetin with selected antibiotics against test strains. On testing bacterial growth inhibition with antibiotics, *K*. *pneumoniae*, *P*. *aeruginosa*, and *Y*. *enterocolitica* showed a maximum of 60.02%, 56.46%, and, 60.79% inhibition against kanamycin, erythromycin, and gentamycin respectively. Synergistic activity of quercetin enhanced the susceptibility of test strains in a dose-dependent manner (Table 2). Upon treatment with 40μg/ml of quercetin, *K*. *pneumoniae*, *P*. *aeruginosa* and *Y*. *enterocolitica* showed 92.21, 92.39 and 90.44% increase in sensitivity towards kanamycin, tetracycline and gentamycin respectively. Fractional inhibitory concentration (FIC) index was calculated for each dose of treatment and different combination of antibiotics. Results showed that 44.44% combinations were found to be synergistic and partial synergistic (FICI ≤ 0.75). The additive effect was found in 33.33% (FICI = 0.76–1.0) of combinations, and 22.22% (FICI = 1–4) combinations have not shown any difference in the microbial sensitivity against tested antibiotics (Table 2).

**Table 2:** Synergistic effect of quercetin with antibiotics against food-borne pathogens.

Bacterial strain	Antibiotics	Growth inhibition (%)	Increase in the sensitivity (%) 5 μg	FICI	Increase in the sensitivity (%) 20 μg	FICI	Increase in the sensitivity (%) 40 μg	FICI
*K*. *pneumoniae*	Erythromycin	25.51 ± 1.80	38.53 ± 1.99	0.33	40.88 ± 0.90	0.58	58.58 ± 1.22	0.91
	Tetracycline	42.7 ± 0.49	73.34 ± 0.7	0.511	85.19 ± 0.49	0.75	89.97 ± 0.62	1.08
	Kanamycin	60.02 ± 0.51	70.39 ± 0.13	0.683	88.95 ± 0.44	0.93	92.21 ± 0.41	1.26
*P*. *aeruginosa*	Erythromycin	56.46 ± 1.33	57.43 ± 1.85	0.583	65.90 ± 1.91	0.83	83.79 ± 1.38	1.16
	Chloramphenicol	36.96 ± 1.83	38.61 ± 1.79	0.413	58.90 ± 1.43	0.66	77.59 ± 1.25	0.99
	Tetracycline	52.00 ± 0.47	62.59 ± 1.63	1.083	88.56 ± 0.40	1.33	92.39 ± 0.15	1.66
*Y*. *enterocolitica*	Gentamycin	60.79 ± 1.23	70.25 ± 1.59	0.61	83.72 ± 1.88	0.94	90.44 ± 1.31	1.38
	Ampicillin	23.59 ± 0.84	34.48 ± 0.44	0.31	59.21 ± 1.02	0.64	70.40 ± 1.34	1.08
	Erythromycin	32.73 ± 0.76	48.78 ± 0.77	0.44	71.51 ± 1.70	0.77	87.64 ± 1.85	1.21

FICI = Fractional Inhibitory Concentration Index; < 0.5 synergy; 0.5–0.75 partial synergy; 0.76–1.0 additive effect; 1–4 indifference; >4 antagonism

### Homology analysis

**Fig 6:**
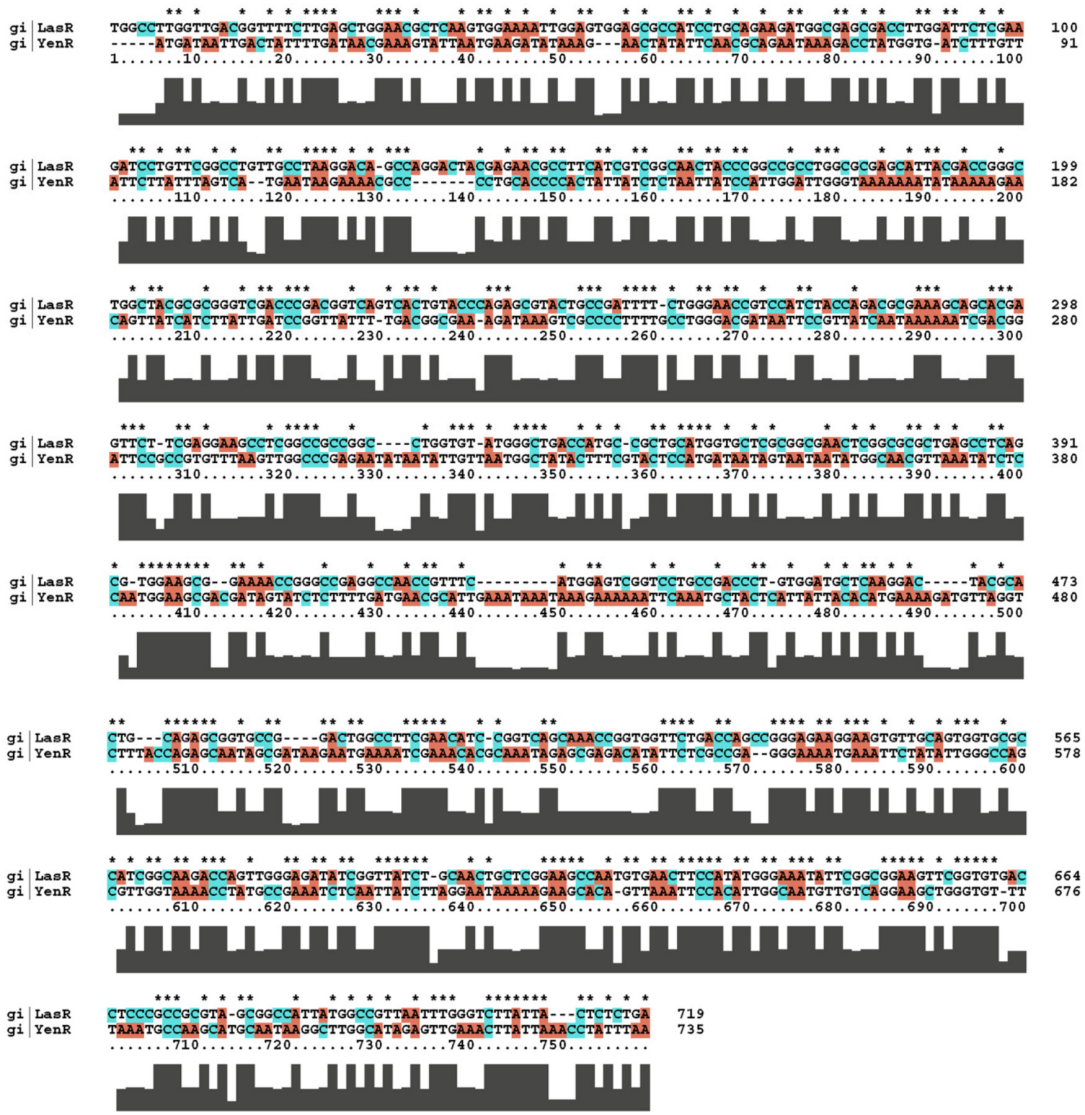
Homology analysis of LasR and YenR gene using clustal W global alignment. Intensity bar shows the similarity between the sequences. *Represents the identical nucleotides.

The position of gene decoding LasR protein in the genome of *P*. *aeruginosa* (EU074852) was found to be 629 to 1348 and the position of YenR gene in *Y*. *enterocolitica* (X76082) was 1547 to 2281. The careful analysis of the image with the use of sequence manipulation suite (http://www.bioinformatics.org/sms2/ident_sim.html) exhibited that both genes shares 60% sequence similarity, and difference of nucleotide is of single nucleotide polymorphism (SNP) type. The intensity of similarity bar shows the similarity between the sequences (Fig 6).

### Docking analysis

The three-dimensional structure of LasR was retrieved from PDB database. Bottomley et al. [22] reported the crystal structure of this receptor protein at 1.80 Å. The NCBI CD database search of this protein reveals that it contains autoinducer domain from residues 20 to 160 which is crucial for the transcription process [23]. PDBSum database was used for its secondary structural component analysis [24]. LasR receptor protein contains 9 α helices, four β strands along with three β hairpin turns. Second β hairpin turn is important as it forms the active site cavity of this protein, refer figure 6a in our earlier publication [25]. This active site was found by ligand explorer toll in PDB database.

**Fig 7:**
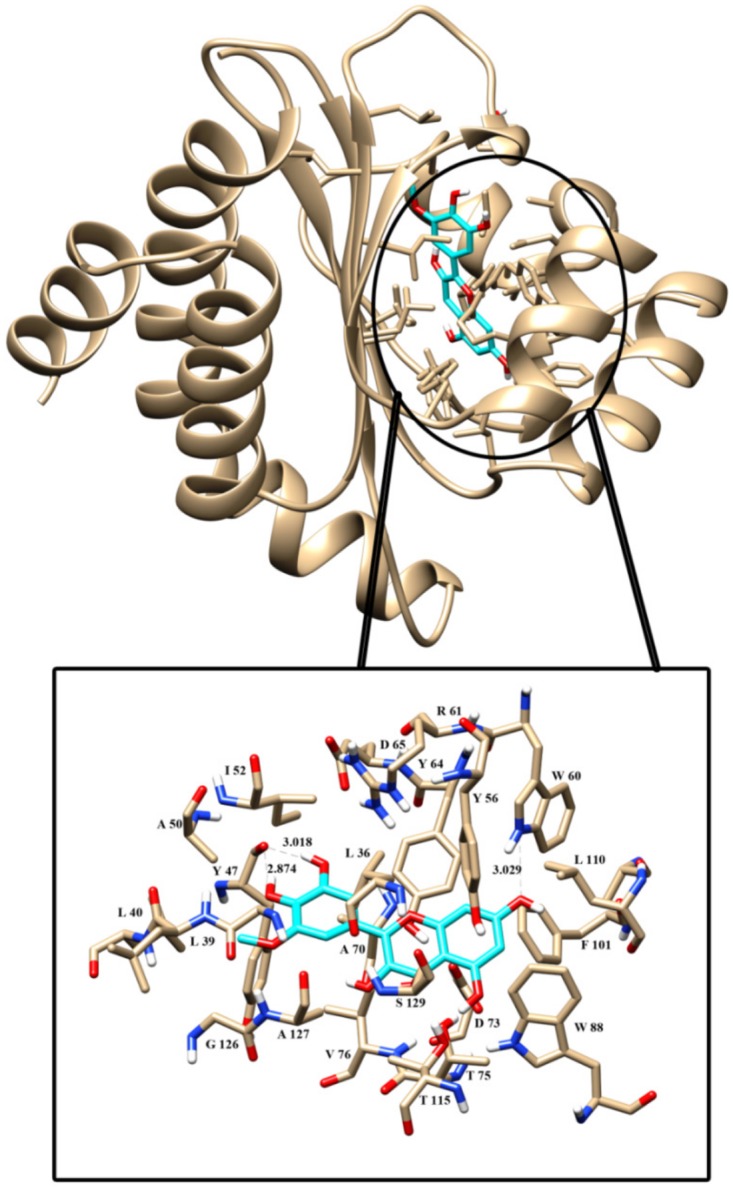
Docked conformation of signaling molecule into the active site of LasR receptor protein. H-Bonds are displayed in dashed line. Residues, which are forming hydrophobic interaction were labeled.

Molecular docking studies were performed to find out the hotspot residues of the protein. These residues are interacting with signaling molecule and active compounds to change its conformation for functioning activity. The dock score of signaling molecule with LasR receptor protein is -4.28Kcal/mol and it was submitted to PDBSum databases for analysis. The LigPlot module of this database helps to visualize the H-bond interaction between residues. Here the numbers of H-bonds are 3 along with 14 hydrophobic interactions. The information of interacting atoms along with the dock score was given in Table 3.

**Table 3:** Details of docked complex of LasR receptor protein with N-octanoyl-DL-homoserine lactone (OHL).

Molecules (Drug Bank ID)	Hydrogen Bonding interactions			Dock score (Kcal/mol)	Glide- Emodel score	Hydrophobic interactions
	Donor	Acceptor	Length (Å)			
N-Octanoyl-DL-homoserine lactone (OHL) 3474204	Lig:: O2 Thr 75:OG1 Asp 73: OD1	Trp 60:NH1 Lig:: N1 Lig:: N1	3.01 3.30 3.00	-4.28	-42.2	Leu 110, Phe 101, Tyr 93, Ala 105, Trp 88, Tyr 56, Ser 129, Tyr 64, Leu 36, Ala 127, Tyr 47, Ala 50, Val 57, Ile52

Quercetin was docked to LasR receptor protein in the second attempt with the same grid parameters used for signaling molecule. After completion of docking, the complex with a maximum dock score -7.20 Kcal/mol was used for further in vitro analysis. This complex was submitted to PDBSum database for further analysis. One H-bond was formed between the protein and active compound along with 15 hydrophobic interactions. The information of interacting atom of protein and active compounds was given in Table 4.

**Table 4:** Interaction of LasR receptor protein with quercetin.

Molecules (Drug Bank ID)	Hydrogen Bonding interactions			Dock score (Kcal/mol)	Glide- Emodel score	Hydrophobic interactions
	Donor	Acceptor	Length (Å)			
Quercetin 5280343	Lig:: O6	Thr 75:OG1	2.64	-7.20	-58.3	Tyr: 64, Tyr: 56, Asp: 73, Ser: 129, Leu: 36, Ala: 127, Val: 76, Ile: 52, Gly: 38, Ala: 50, Gly: 126, Cys: 79, Leu: 40, Leu: 39, Leu: 125

The third iteration of docking was performed to check the competitive nature of quercetin against signaling molecule. All the ligands including signaling molecules docked to the same site that was used in earlier docking. In the presence of signaling molecule, the dock score of docked complexes was changed. In this attempt, the dock score of a signaling molecule was -5.62 Kcal/mol. This complex was submitted to PDBSum database for analysis. From the LigPlot analysis, it was revealed that there were 1 H-bonds formed between LasR and quercetin along with 15 hydrophobic interactions, which provided additional strength to this complex.

The information of all interacting atoms of protein and quercetin along with H-bond direction and distances was given in Table 5. The pose of quercetin in LasR receptor protein was shown in Fig 7. Black dashed line represents the H-bonds.

**Table 5:** Residues of LasR receptor protein interacting with quercetin and N-octanoyl-DL-homoserine lactone (OHL) through various interactions.

Molecules (Drug Bank ID)	Hydrogen Bonding interactions			Dock score (Kcal/mol)	Glide- Emodel score	Hydrophobic interactions
	Donor	Acceptor	Length (Å)			
Quercetin 5280343	Thr: 75 OG1	Lig::O6	2.63	-9.17	-55.2	Leu: 39, Leu 40, Gly 38, Gly 126, Leu 125, Ala 50, Cys 79, Val 76, Ile 52, Leu 36, Asp 73, Ala 127, Ser 129, Tyr 56, Tyr 64

**Fig 8:**
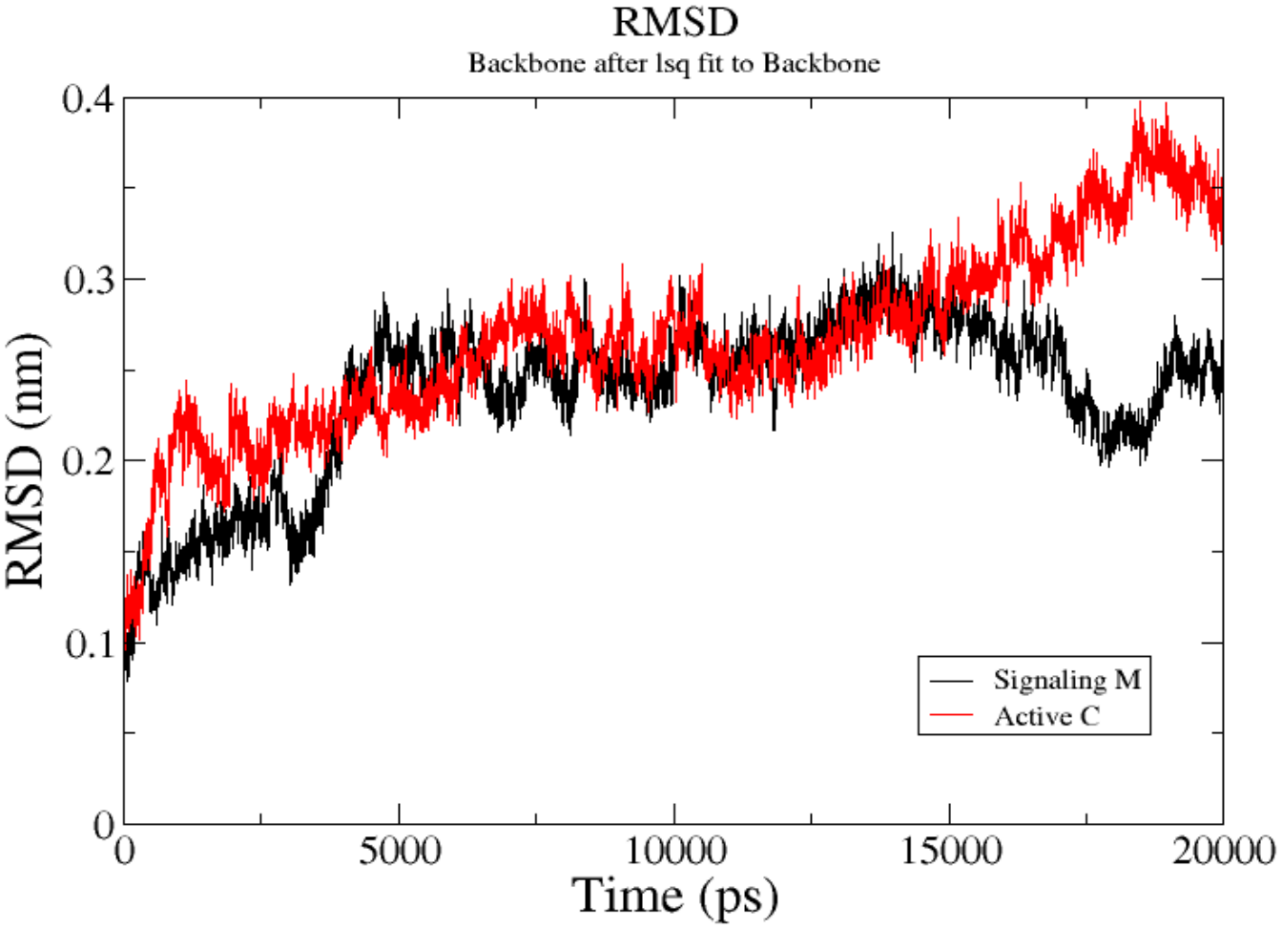
RMSD profile of simulated complexes. Black color line representssignaling molecule complex with LasR while red color line represents the complex of active compound with LasR.

### Molecular dynamics simulation

Molecular dynamics simulation was performed to predict the conformational changes for activation and deactivation of LasR receptor protein in the presence of signaling molecule and quercetin respectively. The simulations were performed with two complexes, LasR-OHL, and LasR-quercetin. The simulations were run for 20 ns with the time step of 2 ps.

The RMSD profile was generated to screen the behavior of protein throughout simulation with signaling molecule and quercetin. It can be seen in the RMSD profile (Fig 8) that the protein-signaling molecule is unstable as compared to protein-test compound complex. This instability was caused because signaling molecule handles the activation of LasR protein. Here the activation takes place due to opening of loop 2 (residues 40–51) and loop 3 (residues 66–80). While in case of the active molecule, it closes activation site by closing this loops.

This opening and closing of the pocket was validated by submitting both the complex protein-signaling molecule and protein-test compound to CASTp server, which calculates the accessibility of protein pocket by using water the molecule as a probe. Here the volume of pocket in the presence of signaling molecule was 307.6 Å3 and in the presence of test compound was 179.8 Å3.

## Discussion

In the current study, quercetin was evaluated by both *in-vitro* and *in-silico* techniques for its potential to hamper QS-regulated phenotypes and biofilm formation in food borne pathogens. Quorum quenching potential of quercetin was initially screened against *C*. *violaceum* CV026 and MTCC 2656. In plate incubation assay, quercetin exhibited a concentration-dependent reduction in violacein production, indicated by the zone of pigmentation loss. In flask incubation assay with CV026, quercetin reduced the violacein production up to 83.23% at the concentration of 80μg/ml. Above results are comparable with those of Zhang et al. [26], who reported 87.56% of violacein inhibition by *Rosa rugosa* at the concentration of 1.20mg/ml in CV026. Vasavi, Arun, &Rekha [27], reported >50% of the reduction in violacein production was observed at the concentration of 100μg/ml of *Centella asiatica*.

Biofilm formation by food-borne bacteria is one of the most important aspects of its pathogenicity. Quorum sensing is one of the crucial factors in the process of biofilm formation [28]. Thereby, interfering with signal-mediated QS system may regulate the biofilm formation. Results obtained in this study showed that quercetin at all tested concentration efficiently reduced the biofilm formation in test pathogens. Our findings are in accordance with those of Packiavathy et al. [29] who reported that the biofilm formation by food-borne pathogens treated with methyl eugenol (10μg/ml) is scarce when compared with that of untreated ones.

Biofilm formation was characterized in part by the production of highly extensive EPS network. EPS production confers facilitation of initial attachment of bacteria; enhanced resistance to antimicrobial agents and environmental stress; formation of microcolony structure [30]. Thus, inhibition of EPS production may facilitate the direct exposure of food borne pathogens to the antibiotics, may in turn facilitate the eradication of biofilm. Here, reduced EPS production was observed in all test pathogens when treated with quercetin. Abraham et al. [31] reported that *Capparis spinosa* reduced EPS production up to 67% in *P*. *mirabilis*. A viscous exopolysaccharide, alginate production serves to protect bacteria from adversity in its environment; enhances adhesion to the surfaces and also protection against human leukocytes. As depicted in Fig 5, quercetin inhibited the alginate production of *P*. *aeruginosa* in a concentration-dependent manner. In an earlier study Owlia, Rassooli, & Saderi, [32] reported inhibition of alginate production in *P*. *aeruginosa* by *Matricaria chamonilla*.

Swimming and swarming motility influence bacterial biofilm formation by urging the surface attachment. It is evident from our findings that quercetin significantly reduced the flagella-mediated motility of test pathogens when compared with control. Results obtained are comparable with Damte et al. [33] who reported 71% inhibition of *Pseudomonas* swarming motility by plant extracts. In one another finding, Niu & Gilbert [34] reported that cinnamaldehyde reduced biofilm formation in *E*.*coli* by inhibiting swarming motility. Hence, quercetin appeared to reduce the biofilm formation in food-borne pathogens by interfering the ability to reach the substratum. Also swimming and swarming motility is considered to be an important virulence factor in bacteria. Here, quercetin exhibited a considerable reduction in the motility of test pathogens.

It was found that the test pathogens were less sensitive to the antibiotics, such as kanamycin, tetracycline, erythromycin, gentamicin, ampicillin, and chloramphenicol. Bjarnsholt et al. [35] proved the mechanism of enhanced susceptibility to antibiotics relies upon signaling mechanism. On treatment with added quercetin, enhanced susceptibility was observed towards all tested antibiotics. Our results revealed that the anti-quorum compound that is non-antibacterial may overcome the resistance by acting synergistically with conventional antibiotics. It was reported that *V*.*vulnifcus* showed enhanced sensitivity towards doxycycline, in a report by Brackman et al. [36].

*In-silico* analysis may prove the mode of action by which the test compounds exhibit the quorum quenching potential. Molecular docking analysis of LasR receptor protein showed that quercetin binds rigidly to the receptor with high docking score when compared with signaling molecule in both docking conditions (signaling molecule docked with and without quercetin). The strong interaction between the compounds may be due to the binding of particular specific groups, which mediates conformational changes in the receptor protein. RMSD profile showed that throughout the simulation, LasR-quercetin complex is more stable than the LasR-signaling molecule complex. In a recent study Mowafy et al. [12] evidenced that docking analysis may suggest the quorum quenching efficiency of test compounds. It was proved through molecular docking studies that aspirin can act as an anti-quorum agent against *P*. *aeruginosa*.

In brief, the present study evidenced that quercetin efficiently inhibited biofilm formation and other QS regulated phenotypes like violacein inhibition, EPS production and alginate production in selected food-borne pathogens. Quorum sensing inhibitors from natural products such as fruits and vegetables are promising sources that can potentially inhibit QS. Quercetin is one of such compound present in fruits, vegetables, nuts, and grains, which are non-toxic to humans and also readily available. *In-silco* studies were evidenced that quercetin bind more rigidly with the receptor protein than the signaling molecule which proves that quercetin may act as a potential competitive inhibitor of signaling molecules towards LasR protein activity. Both *in-vitro* and *in-silco* studies were done to prove the QS-inhibitory activity of quercetin. As all the experiments conducted were at the sub-MIC level, it is not expected to impose pressure on test pathogens to develop resistance, which offers a new hope for combating with multi-antibiotic resistant bacteria. In future animal studies may be conducted to demonstrate the activity of quercetin over pathogenic infections, which may render interesting results.
