# Correction: Quercetin Influences Quorum Sensing in Food Borne Bacteria: *In-Vitro* and *In-Silico* Evidence

**DOI:** 10.1371/journal.pone.0148471

**Published:** 2016-01-29

**Authors:** The PLOS ONE Staff

## Notice of Republication

Two images in the originally published article were duplicated from the following previous publication:

Gopu V, Kothandapani S, Shetty PH. Quorum quenching activity of *Syzygium cumini* (L.) Skeels and its anthocyanin malvidin against *Klebsiella pneumoniae*. Microb Pathog. 2015; 79: 61–69. [2]

The article was republished on January 19, 2016, to replace copyrighted material and address the inclusion of an incorrect image. Please download the article again to view the correct version.

Due to an error, the photograph used to generate Fig 1 of the *PLOS ONE* article is a duplicate of the photograph used in Figure 1 of the *Microbial Pathogenesis* article [2]. The image originally included in the *PLOS ONE* article is incorrect; a revised Fig 1 has now been provided in the republished article.

Additionally, Fig 7 of the *PLOS ONE* article, depicting the three dimensional structure of the LasR receptor protein, was a duplicate of Figure 6a of the *Microbial Pathogenesis* article [2]. This image has been removed, and the previous *Microbial Pathogenesis* figure is cited and discussed in its place.

The authors apologise for these errors.
